# High-Aluminum-Affinity Silica Is a Nanoparticle That Seeds Secondary Aluminosilicate Formation

**DOI:** 10.1371/journal.pone.0084397

**Published:** 2013-12-13

**Authors:** Ravin Jugdaohsingh, Andy Brown, Martin Dietzel, Jonathan J. Powell

**Affiliations:** 1 MRC HNR, Elsie Widdowson Laboratory, Cambridge, United Kingdom; 2 Institute for Materials Research, SPEME, University of Leeds, Leeds, United Kingdom; 3 Institute of Applied Geosciences, Graz University of Technology, Graz, Austria; University of Queensland, Australia

## Abstract

Despite the importance and abundance of aluminosilicates throughout our natural surroundings, their formation at neutral pH is, surprisingly, a matter of considerable debate. From our experiments in dilute aluminum and silica containing solutions (pH ~ 7) we previously identified a silica polymer with an extraordinarily high affinity for aluminium ions (high-aluminum-affinity silica polymer, HSP). Here, further characterization shows that HSP is a colloid of approximately 2.4 nm in diameter with a mean specific surface area of about 1,000 m^2^ g^-1^ and it competes effectively with transferrin for Al(III) binding. Aluminum binding to HSP strongly inhibited its decomposition whilst the reaction rate constant for the formation of the β-silicomolybdic acid complex indicated a diameter between 3.6 and 4.1 nm for these aluminum-containing nanoparticles. Similarly, high resolution microscopic analysis of the air dried aluminum-containing silica colloid solution revealed 3.9 ± 1.3 nm sized crystalline Al-rich silica nanoparticles (ASP) with an estimated Al:Si ratio of between 2 and 3 which is close to the range of secondary aluminosilicates such as imogolite. Thus the high-aluminum-affinity silica polymer is a nanoparticle that seeds early aluminosilicate formation through highly competitive binding of Al(III) ions. In niche environments, especially *in vivo*, this may serve as an alternative mechanism to polyhydroxy Al(III) species binding monomeric silica to form early phase, non-toxic aluminosilicates.

## Introduction

Aluminosilicates are aluminum (Al), silicon (Si) and oxygen bearing minerals often associated with cations such as sodium, potassium and magnesium. They are heterogeneous in structure, ubiquitous in nature and the main components of many soils and sediments [1,2]. Aluminosilicates may act as template-catalysts for organic reactions [3] and such properties may have allowed the very early formation of pre-biotic molecular structures. While this latter point remains speculation, aluminosilicates are nonetheless extensively used in industrial applications as sorbents and solid phase catalysts (e.g. zeolites, smectites and imogolite) [4]. More recently there has been interest in aluminosilicates in physiological systems. The intake of aluminosilicates into the human body, mainly via the lungs and gastrointestinal tract, is an obvious route of exposure [5,6] but, also, the formation of aluminosilicates *in situ* (intracellularly) may occur at least in lower animals [7,8]. Based on several lines of evidence it has been proposed that, in certain multi-cellular organisms, the cellular uptake of Al may lead to the rapid mobilization and accumulation of dissolved silica (e.g. monosilicic acid, which is also referred to as orthosilicic acid) in distinct intracellular compartments (lysosomes), thereby ‘neutralising’ Al ions through the formation of intracellular, non-toxic aluminosilicates [7,8]. Indeed, it is generally recognized that soluble silica limits the toxicity of Al, a potent neurotoxin [9,10], by the formation of non-toxic hydroxyaluminosilicates (HAS [11,12]). This can occur *ex vivo* where it limits the absorption or bioavailability of Al (III) ions [13-15] or *in vivo* where it seems to increase the urinary excretion of Al [11,13,16]. This interaction is highly dependent on the silica concentration and a high Si to Al ratio is required for the amelioration of Al toxicity to be observed [12,15].

How this could occur is unclear as, although the abundance and significance of aluminosilicates throughout natural and synthetic environments are well known, surprisingly, their early formation, especially at low temperature, low concentration and neutral pH, is a matter of considerable debate. Aqueous Al (III) and silica, in their monomeric hydrated forms, have low affinity for each other (log *K*_eff_ 4.7 at pH 7.2, 25°C) and therefore their interactions are of negligible importance in natural waters [12,17]. For these species to first ‘engage’ it has been shown that Al(III) needs to undergo hydrolytic polymerisation, forming aqueous Al poly oxohydroxides, which have sufficient affinity for effective binding of monosilicic acid (Si(OH)_4_) [12,18,19]. *In vivo* this is most unlikely however as metal ions, such as Al(III), are carefully chaperoned by chelators such as citrate and transferrin, to prevent polyoxohydroxy formation. Hence, as an ‘alternative’ mechanism for *in vivo* aluminosilicate formation we (RJ & JJP) previously reported on an aqueous silica polymer with very high affinity for monomeric Al(III) (log *K*_eff_ 11.7 ± 0.03 at pH 7.2, 25°C) [20]. This high-aluminium-affinity silica polymer (HSP), originally termed ‘oligomeric silica’, is transiently stable at Si concentrations below the solubility limit of amorphous silica (<< 2 mM; at 25°C) and its metastability is greatly increased by binding Al(III) ions [20]. HSP and its aquo-complex with aluminum are resistant to degradation in the gastrointestinal tract leading to marked inhibition of aluminum absorption [13]

HSP or similar species are likely to be present in certain environmental niches and *in vivo*, such as in cell compartments and the renal tubules, where Si concentrations may at times exceed the solubility limit of amorphous silica [21]. Indeed, renal calculi have been identified with the long term use of silicate antacids [22]. The formation of HSP or similar species (in the renal tubes) could provide a mechanism for the postulated increased excretion of Al following ingestion of silica-rich water [11,13,16]. The size and structure of HSP and the nature of its interaction with aluminum are thus of particular importance in environments where the formation of Al poly oxohydroxides is limited (e.g. by [Al], ligands and/or pH) as HSP acquisition of Al(III) ions may then drive the initial formation of non-toxic (hydroxy) aluminosilicates. The aim of the present study was to characterise the structure and properties of HSP and its Al-containing counterpart (referred to herein as ASP), using the β-silicomolybdate assay, atomic resolution imaging with scanning transmission electron microscopy (STEM), and fine probe electron energy loss spectroscopy (EELS). Subsequently, we studied Al-binding by HSP in comparison with well-characterised silica colloids using a chelator-based competition binding assay as previously described [20]. Finally, we investigated whether HSP would compete with transferrin for Al. Transferrin is the main, high-affinity, Al chelator in the circulation, and our previous findings [20] implied that HSP may have sufficient Al(III) affinity to compete with this biomolecule.

## Materials and Methods

### Materials

Water used in this study was ultra-high purity (UHP), 18 MΩ cm^-1^, from an Elga water purifier (High Wycombe, UK). Human apo-transferrin protein and 1, 2-dimethyl-3-hydroxy-4-pyridinone (DMHP, 98% purity) were gifts from Dr Robert Evans (Brunel University, UK; [23]) and Dr Mike Stockham (Vitra Pharmaceutical Ltd, UK), respectively. Aluminum nitrate nonahydrate (AnalaR grade), morpholinepropanesulphonic acid (MOPS; Biochemical grade > 99% purity), high purity sodium bicarbonate (Normapur^TM^ AR), hydrochloric acid (5 N, volumetric standard) and sodium hydroxide pellets (AristaR grade; for pH adjustment) were from Merck Ltd (Lutterworth, UK). Sodium silicate stock solution (6.9 M Si in 4.77 M NaOH), nitrilo tri-acetic acid (NTA; 99% purity), Ludox silica colloids SM30 (7 nm nominal diameter; specific surface area 345 m^2^ g^-1^), LS30 (12 nm nominal diameter; 215 m^2^ g^-1^) and TM50 (22 nm nominal diameter; 140 m^2^ g^-1^), sodium hydroxide (1 N, volumetric standard; for pH adjustment) and hydrochloric acid (1 N, volumetric standard; for pH adjustment) were from Aldrich Chemical Co (Gillingham, UK). Polypropylene plastic-ware was used throughout from Merck Ltd and from Medfor Products Ltd (Farnborough, UK). Far-UV silica (quartz) cuvette (10 mm path-length), pH meter (pH checker; Hanna Instruments) and roller mixer (Spiramix 5, Dently) were from Merck Ltd. 

### Preparation of high-aluminum-affinity silica polymer (HSP)

The polymeric silica solution (42 mM Si in 10 mM MOPS buffer, pH 7.2) containing the high-aluminum-affinity silica polymer (HSP) was prepared as previously described [20]. Briefly, an aliquot of the sodium silicate stock solution (6.9 M Si, 4.77 M NaOH) was diluted in UHP water. After thorough mixing (30 min on the roller mixer), MOPS buffer (pH 7.2) was added and after further agitation on the roller mixer the pH was adjusted to 7.2 with 1 N HCl. HSPs are formed within minutes of pH neutralization of the sodium silicate solution and reach maximum affinity for Al(III) at 3 h and maintain this (high Al affinity) for 48 h [20].

### Characterization of HSP and ASP

It is important to emphasise that the concentrations of Si (320 µM) and Al (8 µM) used here limit the use of certain analytical techniques, including solvated particle sizing and NMR where the detection limits are higher.

#### Aluminum binding affinity capacity of HSP compared with commercial silica colloids

Polymeric silica solution containing HSP was prepared as described above and aged for 24 h at room temperature prior to use. 6 mM Si solutions of the Ludox silica colloids were prepared by dilution of the stock basic silicate solutions (≤12 M Si, pH 8-10) in 10 mM MOPS buffer and then pH neutralization with 1 N HCl to pH 7.2. These colloidal silica solutions were used immediately upon dilution and pH neutralization. Stock solutions of Al nitrate (20 mM Al(III), pH 3) and DMHP (20 mM) were also prepared as previously described [20]. A solution containing 0.24 mM 1:1 DMHP:Al complex was then prepared in 10 mM MOPS buffer (pH 7.2) by mixing equal volumes of the Al(III) and DMHP stock solutions. Aliquots of this solution were then titrated with the polymeric silica solution or the commercial Ludox silica colloids of varying mean particle size. The 1:1 DMHP-Al complex (8 µM, 10 mM MOPS pH 7.2) with polymeric silica (0–4 mM) or Ludox silica colloids (0–6 mM Si) were incubated at room temperature for 48 h prior to collection of UV spectra between 230–330 nm, on a LKB Biochrom Ultraspec II spectrophotometer (Pharmacia Biotech, UK), at 25°C and using a 4.5 ml far-UV quartz cuvette. Absorbance at 274 nm was extrapolated and corrected for background interferences by subtracting the absorbance at 320 nm.

#### Competition between HSP and human apotransferrin for aluminum binding

Al(NTA)_2_ (2.1 mM in 50 mM MOPS buffer, pH 7.4) was prepared by mixing aliquots of the stock solutions of Al nitrate nonahydrate (20 mM, pH 2) and NTA (20 mM) in a 1:2 (Al:NTA) ratio in 50 mM MOPS buffer and the pH was adjusted to 7.4. Human apo-transferrin (7–7.69 mg/mL) was prepared by dissolving the solid in 50 mM MOPS buffer (pH 7.4). This solution was stored at 4°C. Sodium bicarbonate (32–33.7 mM) was freshly prepared just prior to use in 50 mM MOPS buffer and pH adjusted to 7.4 with 5 N HCl. Polymeric silica solution (42 mM Si; 10 mM MOPS, pH 7.4) containing HSP was prepared as described above and aged for 24 h at room temperature prior to use. Monosilicic acid (3.75 mM; 10 mM MOPS, pH 7.4) was prepared as previously described [20] and aged for 24 h at room temperature prior to use. Ultra-violet (UV) spectra (220–340 nm) were recorded on a Cary 300 UV/Vis spectrophotometer (Version 9; Varian) using a scan rate of 100 nm/min and data collection interval of 0.167 nm. 

To determine the amount of Al(NTA)_2_ required to get saturated binding of apo-transferrin with Al(III), human apo-transferrin (1.28 mg/mL or ~ 16.31 μM, in 25 mM sodium bicarbonate and 50 mM MOPS buffer, pH 7.4), in the cleaned quartz cuvette, was titrated with three mole equivalents of Al(NTA)_2_. Spectra were recorded between 220–340 nm using 1.28 mg/l apotransferrin in 25 mM sodium bicarbonate and 50 mM MOPS buffer (pH 7.4) as reference. The complete experiment was repeated in triplicate.

A stock solution of the Al-transferrin complex (43 μM Al(III) and 1.38 mg/mL (or 17.6 μM) apotransferrin) was prepared by mixing aliquots of the stock solutions of human apo-transferrin and Al(NTA)_2_ in 25 mM sodium bicarbonate and 50 mM MOPS buffer (pH 7.4). Aliquots of the Al-transferrin complex were then titrated separately with polymeric and monomeric silica solutions (0–3 mM Si, pH 7.4). Solutions were equilibrated at room temperature for 2 h (on a roller mixer, 50 rpm) before collection of UV spectrum between 220–340 nm using apo-transferrin (1.28 mg/mL) in 25 mM sodium bicarbonate and 50 mM MOPS buffer (pH 7.44). [Longer and shorter equilibration periods were investigated and 2 h was deemed most suitable.] The complete experiment was repeated in triplicate.

#### Characterization of HSP and ASP by electron microscopy

Polymeric silica solution (42 mM Si at pH 7.2) containing HSP, was prepared as described above but without MOPS buffer and aged for 3 h at room temperature. Aluminum nitrate (20 mM, pH 3) was prepared as previously described from the Al nitrate nonahydrate salt [20]. An aliquot of the polymeric silica solution was then mixed with 8 μM Al nitrate (pH 3) and pH adjusted to 7.2. The solution mixture (320 µM Si & 8 μM added Al(III)) was aged for 48 h at room temperature, after which a drop was air dried on TEM copper grids coated with a holey carbon support (Agar Scientific, UK). A drop of the diluted, HSP-only containing solution (320 µM Si) without added Al(III) was air-dried on similar holey carbon-coated TEM grids. 

TEM investigations were carried out using a Philips/FEI CM200 field emission gun (FEG)-TEM operating at 197 keV and fitted with a Gatan Imaging Filter (GIF 200) and Oxford Instruments UTW ISIS X-ray detector (EDX). Bright field images were digitally acquired with a CCD camera with 1024 × 1024 pixels. Bulk energy loss spectra were acquired in diffraction mode (image coupled) from an area defined by the smallest SAED aperture (approximately 180 nm in diameter at the image plane), with a collection semi-angle of 6 mrad and a convergence semi-angle of approximately 1.5 mrad. Electron energy loss energy-filtered (elemental) mapping of the aluminosilicates was carried out with a 5 eV energy selecting slit and an objective aperture inserted (~3 mrad collection semi-angle).

The STEM work was performed using an aberration-corrected dedicated STEM (SuperSTEM 1, Daresbury Laboratories, UK) with cold field emission filament operating at 100 keV ± 0.3 eV. The microscope was equipped with a Gatan Enfina EEL spectrometer, a CCD camera, and Digital Micrograph (DM) 3.9.3 with Gatan Microscopy Suite (GMS) 1.4.3 used for scan control and data acquisition. SuperSTEM 1 combines a base microscope, namely a VG HB 501 FEG-STEM, with a NION^TM^ C_s_ corrector. The electron beam convergence semi-angle was 24 mrad and the collection semi-angle was 19 mrad. The high angle annular dark field (HAADF) detector gathers electrons scattered through a semi-angle range of 70 to 210 mrad. The minimum probe size typically obtained with this microscope is 1.3 Å. High resolution HAADF images (1024 × 1024 pixels) were digitally acquired using a Gatan Digiscan unit for beam control and the photomultiplier of a modified Vacuum Generators HAADF detector for signal collection. Electron energy loss spectra and energy-filtered (elemental) maps were recorded using the Spectrum Imaging Technique. 

#### Characterization of ASP formation by reaction rate method

The interaction between aluminum and HSP to form Al-rich silica nanoparticles (ASP) was investigated by the molybdic acid reaction rate method. Soluble silica reacts with molybdic acid to form a colored β-silicomolybdic acid complex [19,24]. The formation of this colored β-silicomolybdic acid complex follows a pseudo first order reaction for both monomeric and polymeric silica. The reaction rate is two to three orders of magnitude higher for monomeric silica (reaction rate *k*_m_ = 2 min^-1^) compared to the polymeric silica (reaction rate *k*_p_). In addition, the lower the value of k_p_ the higher the polymerization degree of the polymeric silica [19,24]. 

A polymeric silica solution containing HSP was prepared as described above and aged for 4 h. An aliquot of this HSP solution was then diluted in 8 μM Al(III) nitrate (pH 3) and pH adjusted to 7.2 at room temperature. The solution mixture (370 µM Si and 8 μM Al(III), pH 7.2) was analysed for polymeric and monomeric silica content and the polymerization degree of the silica over a 68-hour period of ageing. 

Development of the β-silicomolybdic acid complex was measured at 390 nm with an UV/VIS-Spectrometer (Varian, Cary 100) over a period of 10 min. The program SICALC was used to estimate the monomer and polymer silica content of the solution as a bimodal size distribution from the recorded data and the total Si concentration [24]. Total concentration of silica was analyzed in 2% HNO_3_ acidified samples by ICP-OES (Perkin Elmer, 4300 DV).

## Results

### Characterization of the Al-rich silica polymer (ASP) by reaction rate method

 The polymeric silica content and polymerization degree of ASP (i.e. in the Al-containing HSP solution) were analysed by the β-silicomolybdate reaction rate method as a function of time after preparation and for up to 68 h. The polymeric silica content of the solution remained constant at 83 ± 2 mol% Si of total dissolved silica within the analytical uncertainty of about ± 5 mol%. The remaining silica, 17 mol% Si, was related to monomeric silica content. Thus no measurable decomposition of ASP with respect to polymeric silica content occurred during the 68 h period, confirming our previous observations [20]. The reaction rate, *k*_p_, ranged from 55.83 to 85.2 ms^-1^ with a mean value of 67.8 ± 10.2 ms^-1^ (i.e., within an analytical uncertainty of about 15%), suggesting that the polymerization degree is in the range of high polymeric species (Table 1) [25], so TEM/STEM were used for further characterisation (as noted above, particle sizing and NMR are insufficiently sensitive to characterise Al/Si at these dilute concentrations).

**Table 1:** Characterization of ASP: polymeric silica content and reaction rate constant (*k*_p_) for the formation of the β-silicomolybdic acid complex as a function of time after preparation.

**Time (h)**	Polymeric silica content* (mol % Si)	**Reaction rate constant (*k*_p_) (ms^-1^)**
0	84	58.8
1	83	70.8
3	83	66.6
20	83	85.2
44	83	70.2
68	80	56.8

^*^ With respect to total dissolved silica (0.256 mM Si)

### Characterization of HSP and ASP by electron microscopy

**Figure 1:**
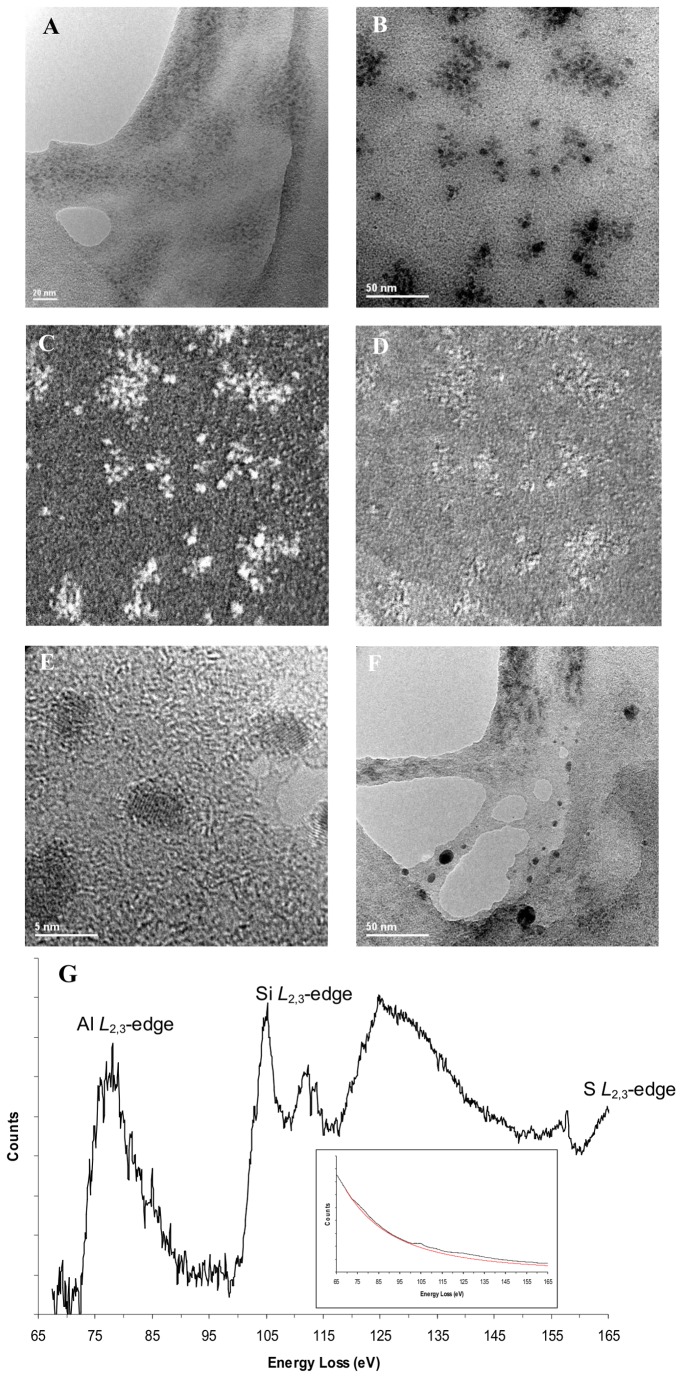
TEM analysis of the Al-containing high Al-affinity silica polymer. Transmission electron microscope analysis of air-dried, 48 h aged, dilute Al and high Al affinity silica polymer containing-solution (8 µM Al(III), 320 µM Si, pH 7.2) showed an amorphous silica gel with dark particulates under low (A) and high magnification (B) bright field (BF) TEM. Electron energy loss spectroscopy (EELS) energy-filtered elemental mapping showed these dark particulates to be Al-rich (C) and co-localised with Si (D). [The magnification in C & D is the same as in B.] Aluminum was confined to the particulates and absent from the gel. The shape of the background stripped Al and Si *L*_2,3_-EELS edges from a region of gel and particulates also suggested Al and Si to be in tetrahedral coordination (G and REF). Insert is the as-recorded spectrum (black) and a typical background estimate (red) used to extract the edges. Higher resolution imaging showed these Al-rich particulates to be ~ 4 nm in size and crystalline with the atomic fringes clearly visible (E). Care was taken to minimize the electron fluence (electrons nm^-2^) since irradiation with a high fluence of the high energy electron beam, leads to damage of the sample and results in agglomeration to produce 20 nm, particulates (F).

 TEM analyses of the air-dried, dilute ASP mixture (8 μM Al(III) and 320 µM Si, pH 7.2) revealed an amorphous silica gel containing particulate matter (Figure 1A & B). High resolution imaging indicated a mean diameter of 3.9 ± 1.3 nm (range: 2.5–7 nm; n=6), with a crystalline structure to these (ASP) particles, and hence the dark contrast relative to the gel (Figure 1E), with atomic lattice fringes being readily observed (Figure 1E). Energy Dispersive X-ray Spectroscopy (EDX) and Electron Energy Loss Spectroscopy (EELS) confirmed the presence of Al, Si and O in the samples. The element-characteristic electron density maps generated by EELS energy-filtering showed that these dark particulates were Al-rich and co-localised with Si (Figure 1C & D). Additionally, Al was clearly confined to the particulates and not associated with the amorphous gel (Figure 1C). The shape of the Al and Si *L*_2,3_-EELS edges lack sharp intensity at the edge onsets suggesting that Al and Si are mostly tetrahedrally coordinated (presumably with oxygen; Figure 1G) [26]. However, the Al:Si ratio of the particulates could not be determined due to the lack of spatial resolution of the image-coupled EELS acquisition method employed here. Namely the high resolution TEM analyses of the Al-rich silica particulates were from a region 185 nm in diameter containing both amorphous silica gel and particles. Thus quantitation of the Al:Si ratio of the particles and gel would greatly overestimate the Si content of the particles. Moreover, even with targeted analysis there is some uncertainty in this ratio due to the difficult estimate of the background subtraction of the overlapping Al and Si L-edges. 

 It should be emphasised that careful attention was paid to avoiding/minimising beam damage as we noted that, at high intensity, the high energy beam used in these analyses could lead to damage of the sample and the artefactual formation of large (~ 20 nm) Al-containing particulates with some Al in octahedral coordination (Figure 1F); based on the increase in intensity at the edge onset of the Al *L*_2,3_ – EELS edge (data not shown). Analysis of the corresponding air-dried, diluted, HSP-only containing solution (320 µM Si without added Al(III) at pH 7.2) revealed a mottled pattern of fine nanoparticles < 5 nm, and a chiefly amorphous appearance albeit with occasional indication of crystallinity (Figure S1). EDX analysis confirmed the presence of Si, O and the absence of Al. Analysis of the Si *L*_2,3_ edge was consistent with tetrahedral co-ordinated Si. 

**Figure 2:**
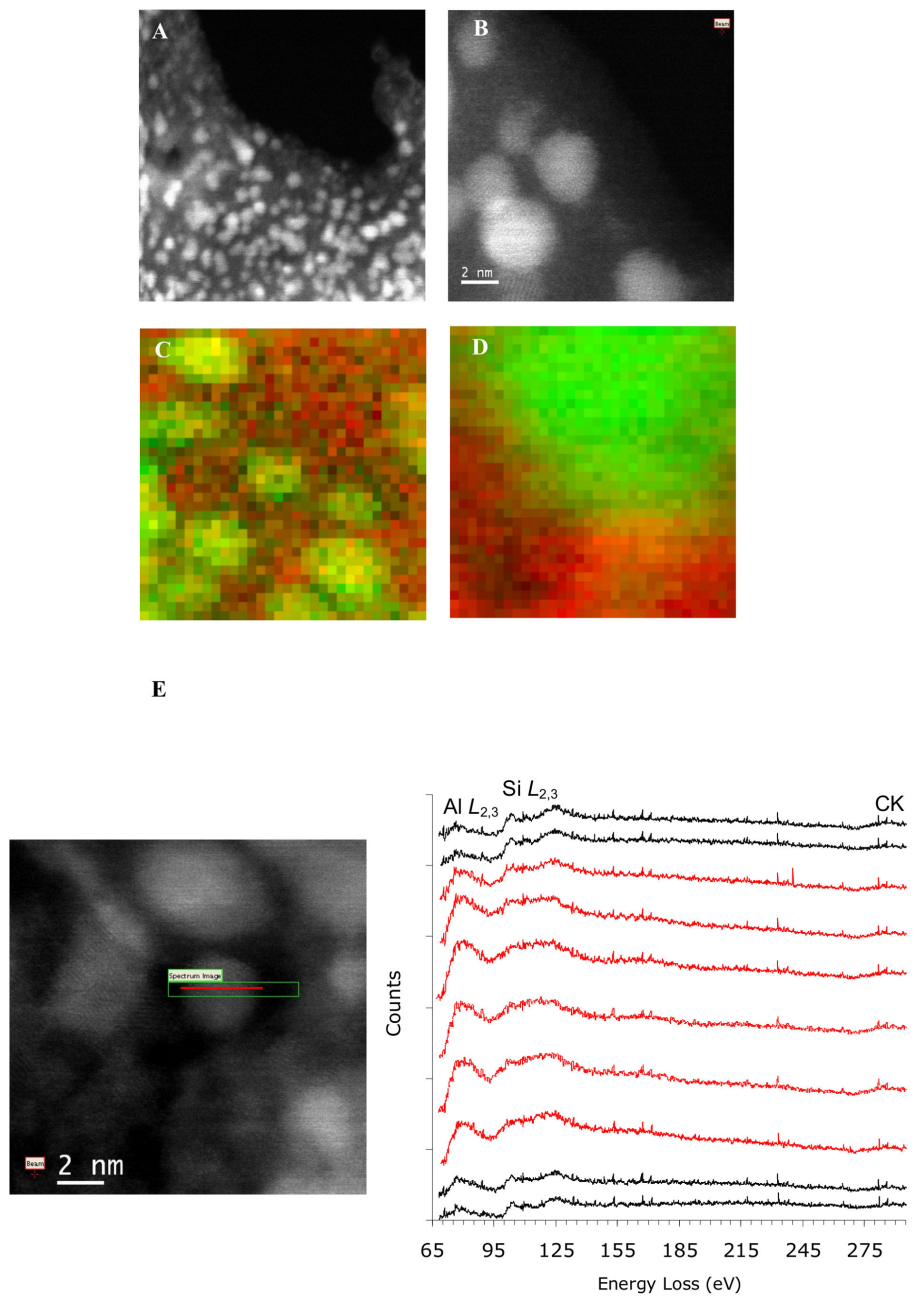
HAADF imaging of the Al-containing high Al-affinity silica polymer. Higher resolution high angle annular dark field (HAADF) imaging of the air-dried, 48-h aged, dilute Al(III) and the high Al affinity silica polymer containing-solution (8 µM Al(III), 320 µM Si, pH 7.2) by aberration corrected scanning transmission electron microscopy (SuperSTEM) confirmed the presence of an amorphous gel with ~ 4 nm particulates (A) that are crystalline in nature (B). (C & D) Show false colour elemental maps where the Al (*L*_2,3_-edge) EELS elemental map has been colored green and the Si (*L*_2,3_-edge) has been colored red and the two overlaid. From this it is clear that Al is confined to the particulates and there is little or no Al in the gel. (E) Shows a series of Al and Si *L*_*2,3*_-edges obtained following a line-scan across a particle (inserted image), confirming little Al in the gel (black line spectra), higher levels in the particle and the presence of Si in the particle (spectra on the particle are colored red).

**Figure 3:**
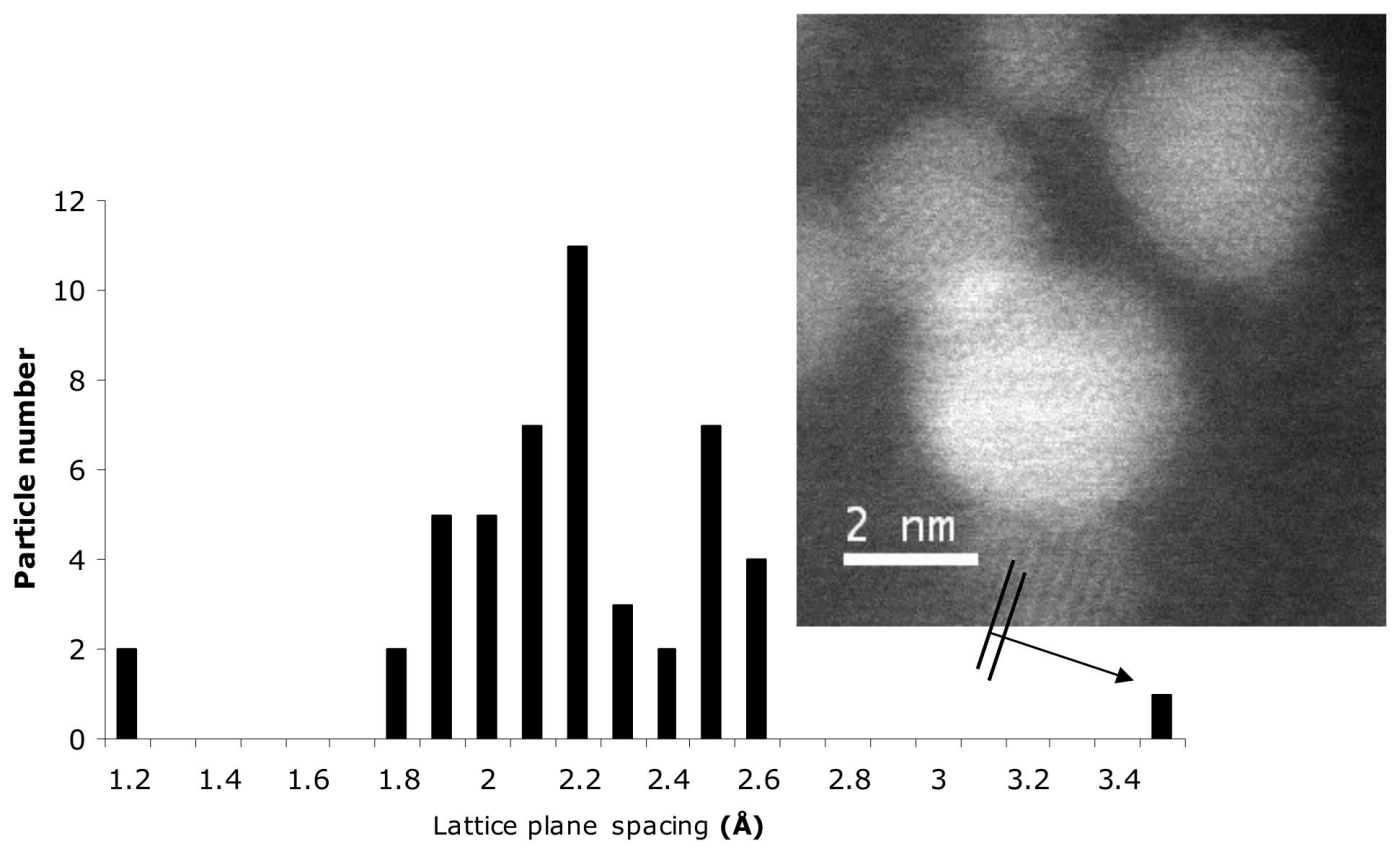
Atomic lattice spacing of the Al-rich silica-containing nanoparticles. A histogram of lattice plane spacing measured (to the nearest 0.1 Å) from 49 individual crystallites (Al rich silica-containing nanoparticles; ASP) in 12 HAADF-STEM and BF-TEM images. Insert: shows measurement of the lattice spacing in a STEM image (from Figure 5B). Comparison of measured lattice spacing to standard X-ray diffraction data, taken from the International Crystal Diffraction Database (ICDD), produced no clear match to a specific phase (see text).

 Aberration corrected STEM (or Super-STEM), with its increased resolution and analytical capabilities (1.3 Å probe) was then used for more accurate, targeted analyses of the ASP. Analyses confirmed an amorphous gel with crystalline nano-particulates approximately 3.7 ± 1.0 nm in diameter (range: 2.6–6.7 nm; n=7; Figure 2A & B). Again, aluminum was consistently found to be confined to the crystalline particulates and the shape of the Al *L*_2,3_-EELS edge suggested Al to be present in tetrahedral coordination (Figure 2C-E) [26]. Quantification of the EELs spectra estimated the Al:Si ratio of the particles to be between 1.6 and 3.2 (average 2.4): the relatively broad range being partly governed by the sensitivity to the background subtraction used for these inherently weak signal-to-background ionization edges. Atomic lattice spacing (d-spacing), visible in images of the individual crystalline nanoparticulates, was measured and varied between 1.8 and 2.7 Å (Figure 3). 

 Since these data suggested surface Al binding by very small silica clusters/nanoparticles we, therefore, next calibrated Al(III) binding of HSP against silica nanoparticles of known sizes.

### Aluminum-binding by HSP compared with commercial silica colloids

**Figure 4:**
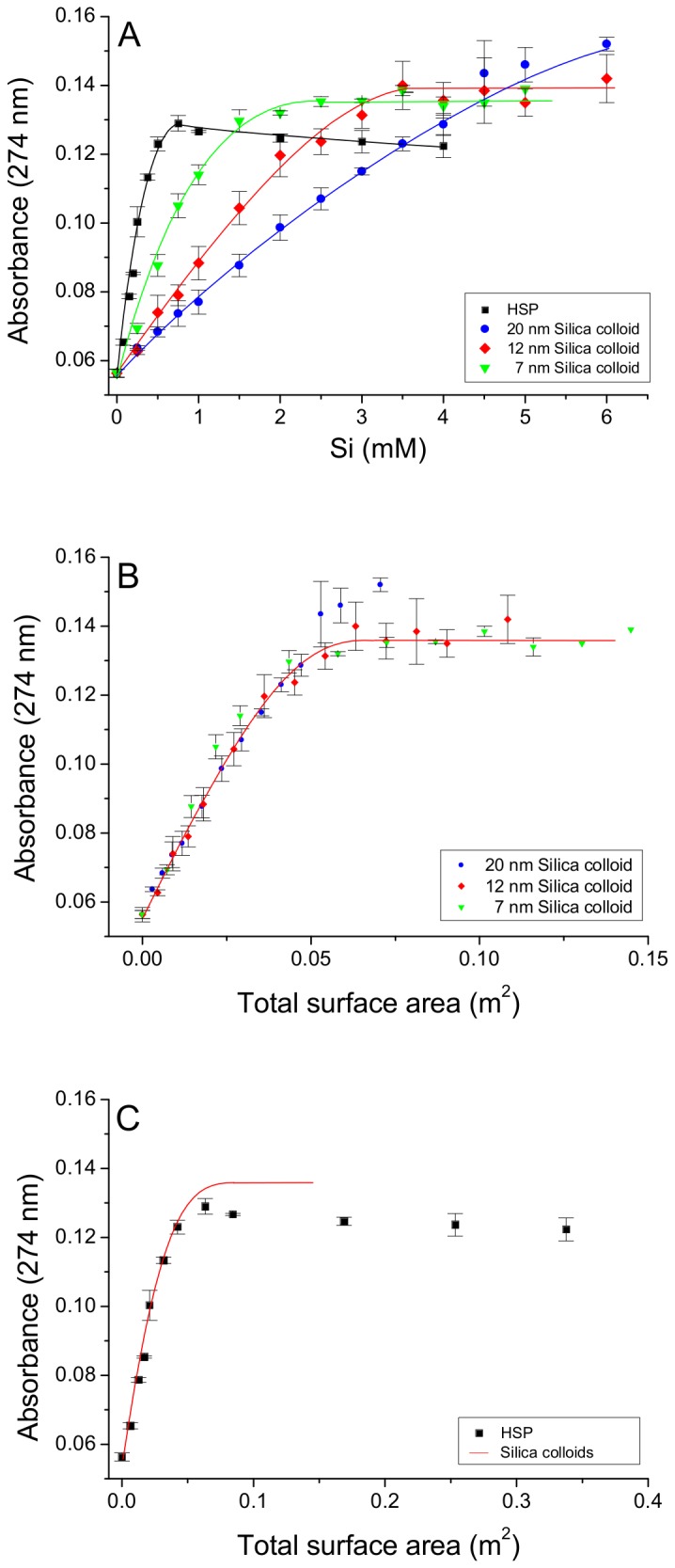
Comparison of Al binding affinity of the high-Al affinity silica polymer with commercial Ludox colloids. (A) Competitive binding of Al(III), from the DMHP-Al complex (8 µM, pH 7.2), by the high Al affinity silica polymer (HSP) and the commercial Ludox silica colloids was assessed at 274 nm by measuring the amount of free DMHP liberated in solution. These results suggest a relationship between Al binding affinity and particle size as SM30 (7 nm) had higher/greater affinity than LS30 (12 nm), which in turn had greater affinity than TM50 (22 nm). (B) To correct for differences in particle size, the Al binding curves for the Ludox silica colloids were re-plotted in terms of total surface area. The red line shows the best fit through the data points. From (A) it was estimated that our polymeric silica was 2.91 times smaller than the smallest Ludox colloid, SM30 (7 nm). This estimate for polymeric silica was confirmed in (C), upon re-plotting the Al binding curve in terms of surface area (squares). The red line shows the best fit line for the Ludox silica colloids (as shown in (B)). Results are the mean (± SD) of two experiments in triplicate.

The DMHP-Al competition assay confirmed our previous findings that HSP competes effectively with DMHP (log *K*_eff_ 9.7 ± 0.03 at pH 7.2 , 0.1 M KCl and 25°C) for aluminum (Figure 4A). At total Si concentrations of ~ 0.75 mM all of the Al(III) was displaced from the DMHP-Al complex and was bound to HSP. In comparison, the commercial Ludox silica colloids (7–22 nm in diameter) competed less effectively and higher concentrations (≥ 2.5 mM Si) were required to displace Al(III) from the DMHP-Al complex (Figure 4A). A trend with mean particle size (or specific surface area) was obtained, such that the smaller the particle the greater the Al binding. Indeed, when the Al binding curves of the three Ludox silica colloids (in Figure 4A) were re-plotted in terms of specific surface area, rather than Si concentration, the three curves overlaid each other (Figure 4B). Assuming similar active Al(III) binding sites, these results suggested that the HSP has a higher specific surface area, and thus a smaller mean particle size, than all three of the Ludox silica colloids investigated, including SM30 the smallest Ludox silica colloid (7 nm in diameter; 345 m^2^ g^-1^). It was estimated from Figure 4A, by comparing the start of the plateau phases (i.e. the concentration of Si required to achieve complete binding of Al by the silica colloids; 0.78 and 2.29 mM, respectively for HSP and SM30), that HSP is 2.9 times smaller in diameter than SM30 (i.e. HSP has a mean particle diameter of 2.4 nm and a specific surface area of about 1,000 m^2^ g^-1^). Hence, when the Al(III) binding curve of HSP was re-plotted using the estimate of its specific surface area, it overlaid the Al(III) binding curve obtained for the three Ludox silica colloids (Figure 4C). 

Our data confirms that HSP competes effectively for Al(III) with a log *K*_eff_ > 9.7 at pH 7.2 (i.e. > Al(III) binding than DMHP), and estimated to be 11.7 at pH 7.2 and 25°C in our previous studies [20], and whilst we do not know what biological binding sites HSP could potentially compete for, transferrin has the highest known Al(III) binding *in vivo*. Hence, our further studies investigated competition for Al(III) binding between these species.

### Competition between HSP and human apotransferrin for aluminum binding

**Figure 5:**
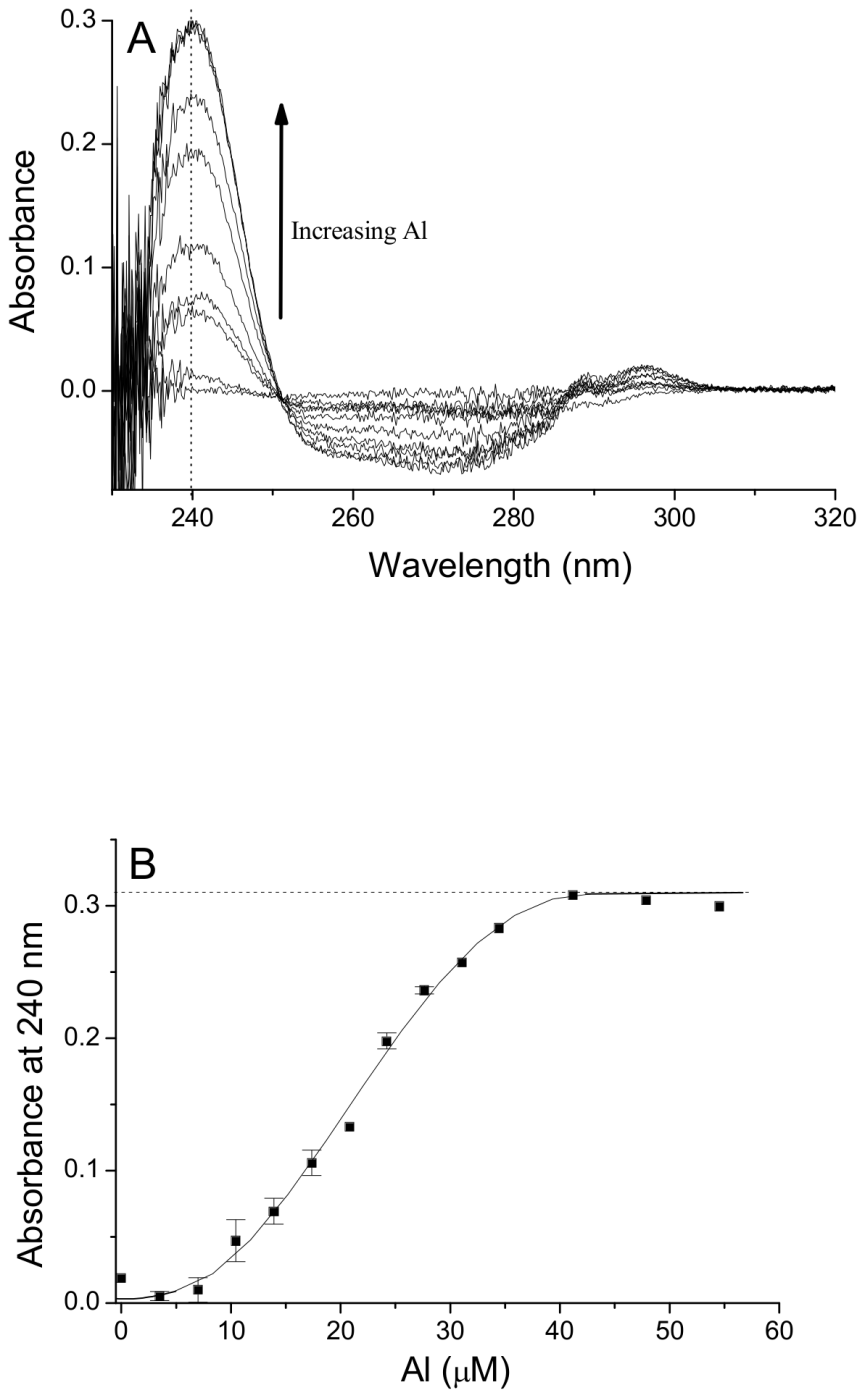
Aluminum binding by human apotransferrin. (A) UV absorption spectra (220–320 nm) of human apo-transferrin (1.28 mg/mL) in 25 mM sodium bicarbonate and 50 mM MOPS buffer (pH 7.4), titrated with increasing concentrations of Al(III) in the form of Al(NTA)_2_. The appearance of a peak (absorbance maxima) at 240 nm was obtained with increasing concentrations of Al(III). A lesser peak between 285–300 nm and a trough between 250–285 nm were also present at the higher Al(III) concentrations. 1.28 mg/mL human apo-transferrin in 25 mM sodium bicarbonate and 50 mM MOPS buffer (pH 7.4) was used as reference. (B) The absorbance maxima at 240 nm was extracted (from A), corrected for variation in baseline/background at 320 nm and plotted against Al(III) concentration. Results are the mean (± SD) of three experiments. Dotted line shows the calculated point of transferrin saturation.

**Figure 6:**
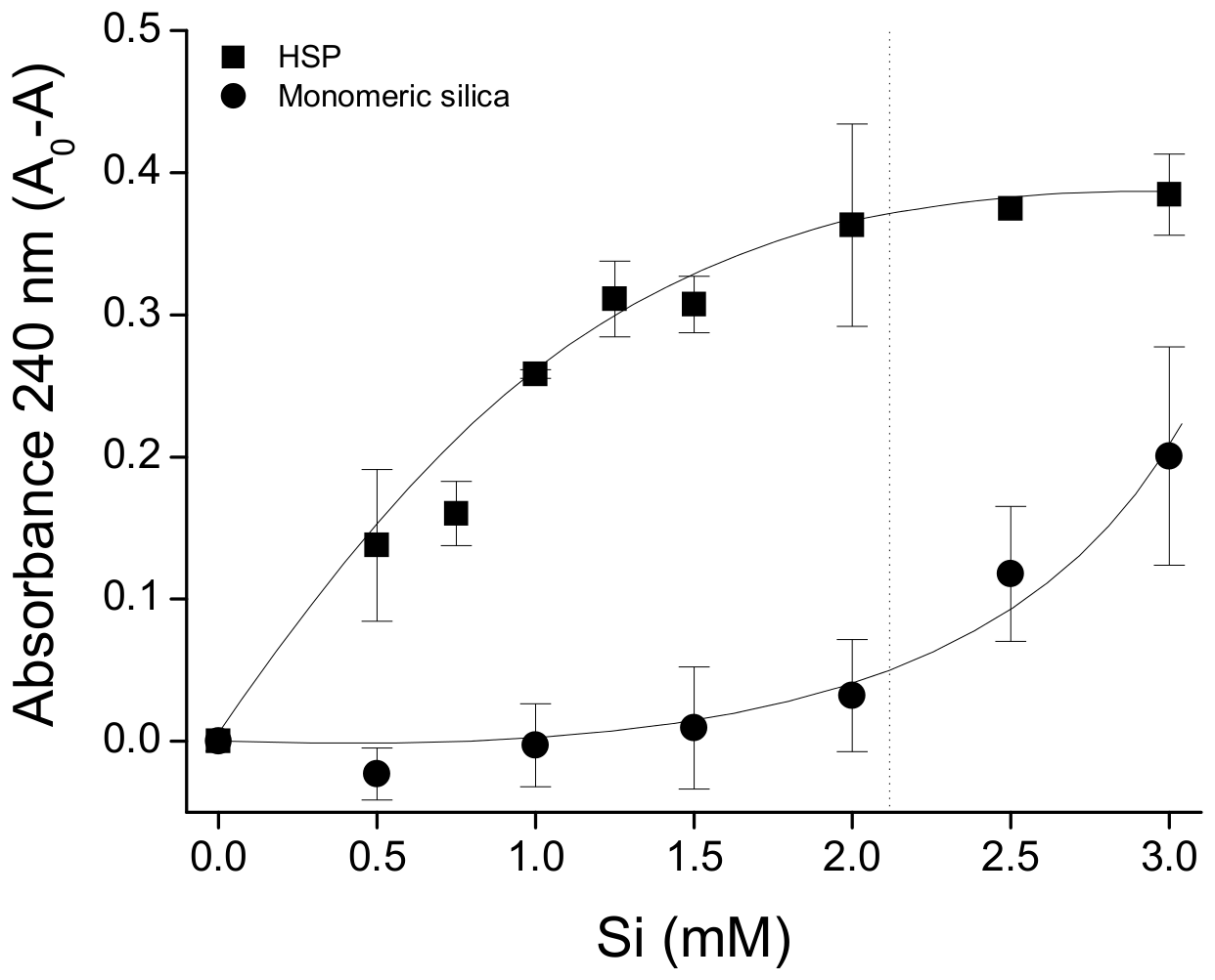
Competition between high-Al affinity silica polymer and human apotransferrin for Al binding. Competition studies with human apotransferrin. The Al-transferrin complex (40 µM Al(III) and 1.28 mg/mL apo-transferrin) was titrated with the high Al affinity silica polymer (HSP, squares) and monomeric silica (circles) at pH 7.4 (25 mM sodium bicarbonate and 50 mM MOPS buffer). The concentration of Al-transferrin complex in solution was measured at its absorbance maxima at 240 nm and corrected for variation in baseline/background at 320 nm. To determine the amount of Al(III) displaced and bound by HSP or monomeric silica, the concentration of Al-transferrin complex in solution at the different silica concentrations (‘A’) was subtracted from the initial/starting concentration of the Al_2_-transferrin complex in solution (A_0_). Results are mean ± SD of three experiments. The dotted line indicates the onset of silica polymerization [14].

Transferrin, the iron-binding (and other metal-binding) transport protein, also has high affinity for aluminium (log *K*_eff_ 11.7–12.2 at pH 7.4) [27] with specific binding sites at the N and C terminus and a degree of non-specific surface binding. Based upon the affinity of HSP for Al(III) from our previous work [20], HSP should compete effectively with transferrin for Al(III) binding. Thus human apotransferrin (1.28 mg/ml) was titrated with Al(NTA)_2_ at pH 7.4 and we confirmed that 2.53 moles of Al(III) were required per mole of apotransferrin to ensure saturated binding (Figure 5), in agreement with other published studies [27-30]. The shape of the curve (Figure 5B) indicates that Al(III) occupies both of the binding sites of human apo-transferrin. This (Al)_2.5_-transferrin complex was then titrated with monomeric or polymeric silica (0–3 mM Si, pH 7.4, 25°C). HSP competed effectively with apo-transferrin for Al(III), and at 2 mM total Si it completely displaced Al(III) from the (Al)_2.5_-transferrin complex (Figure 6). In comparison, monomeric silica barely competed for Al(III) at concentrations < 2 mM Si (Figure 6). However at higher concentrations (> 2 mM Si), ‘monomeric silica’ competed more effectively with apotransferrin (Figure 6) presumably due to its polymerization, and some formation of HSP, which occurs above this critical solubility level of (~ 2 mM) amorphous silica [19].

## Discussion

Previously, characterization of the high-Al-affinity silica polymer (HSP), revealed a silica ‘oligomer’ with fewer than 35 Si atoms and with a log *K*_eff_ for Al(III), at pH 7.2, of at least one million times greater than monomeric silica [20]. This silica oligomer competed effectively for Al(III) with the endogenous chelator, citrate. Furthermore, binding of Al(III) stabilised the dilute silica oligomer to well below the silica de-polymerisation boundary (<< 2 mM total Si) [20]. In this work, our comparative studies with specifically-sized silica particles, which were supported by electron microscopy data, reveal that the silica ‘oligomer’ is a nanoparticle of ~ 2.5 nm diameter. We also confirm its high affinity for Al(III) and show that, under the dilute conditions described, 83% remains nanoparticulate (i.e. stabilized by Al(III)) for > 68 hours, in equilibrium with monomeric silica (Si(OH)_4_). Moreover, we also now show that HSP competes effectively with transferrin for Al(III) and completely displaces Al(III) from the Al-transferrin complex (~ 17.6 μM) at total Si concentrations ~ 1.5 mM. Finally, our data indicate that HSP’s interaction with Al(III) results in the formation of crystalline Al-rich silica nanoparticles (ASP) with a mean diameter of approximately 4 nm, an estimated Al:Si ratio between 2 and 3 and an atomic lattice spacing between 1.8 and 2.7 Å. The evidence that HSP and its reaction product with Al(III) (ASP) are fine nano-sized colloids may also explain the lack of absorption of HSP and ASP in the human gastrointestinal tract [13], which was in marked contrast to soluble silica (Si(OH)_4_)

Our analyses thus clearly confirm the high affinity of the HSP for Al(III). Previously [20], a log *K*_eff_ of 11.70 ± 0.30 (at pH 7.2) was calculated for the interaction of HSP with Al(III), which is similar to the binding of Al(III) by human apo-transferrin (log *K*_eff_ 11.7 and 12.2 at pH 7.4) [27] and thus explains the ease and ability of HSP to compete with human apo-transferrin for Al(III). Complete binding of 43 μM Al(III) by HSP was observed at ~ 1.5–2 mM total Si which is a much higher concentration than the 17.6 μM transferrin. However, the HSP that competes for and binds Al(III), is only a small fraction of the total Si in the polymeric silica solution (about 1%, see below) - TEM/STEM studies here showed that much of the polymeric silica within ASP occurs as an amorphous gel with little associated Al(III) binding – so estimates can only be made based upon bound Al:Si ratios. At 320 µM total Si, the bound Al(III) results in an average Al:Si ratio of 2.4:1 (STEM data), suggesting that only 3.33 μM Si (1.04% to the total Si) was associated with the 8 μM Al(III) (Figure 1).

The re-dissolution of HSP is greatly reduced by binding trace amounts of Al(III) to form ASP. It is known that soluble Al(III) inhibits the decomposition of polymeric silica and reduces the solubility of amorphous silica in water [25,31]. Aluminum adsorbs onto the silica surface and prevents attack by hydroxyl ions [25,32]. Here, the addition of very low concentrations of Al(III) (8 µM) inhibited the decomposition (depolymerisation) of the HSP at pH 7.2 and at [Si] << 2 mM (i.e. below the solubility limit for amorphous silica). The polymeric silica content (83 ± 2 mol % Si of total dissolved silica) remained unchanged through the 68 h period after preparation. It is important to note that in the absence of Al(III), 94% of the polymeric silica would have depolymerised to monosilicic acid within only one hour (estimated by DEPO at 25°C, pH 7.2) [33]. The reaction rate constant *k*_p_ for the formation of the β-silicomolybdic acid complex from ASP also remained unchanged during the 68 h analysis period, suggesting that in the presence of Al(III), the typical condensation reaction with respect to silica does not occur. For “pure” silica species, *k*_P_ can be related to an average particle diameter (d in nm) by the following equation [25]: log(d) = - 0.284 log(*k*_P_) - 0.252. This approach results in an average diameter for ASP between 3.6 to 4.1 nm: i.e. comparable to values obtained here by TEM and STEM. 

The estimated Al:Si ratio of 2 to 3 for ASP suggests the formation of secondary aluminosilicates such as imogolite. A distinct aluminosilicate phase was however not identified here by comparison with the X-ray diffraction pattern of potential precipitates such as imogolite and allophane. Wilson et al. [34], identified, by FT-IR and NMR (^29^Si and ^27^Al), a mixture of protoimogolite and disordered allophane in aluminosilicate preparations prepared at pH 6 by mixing Al and Si solutions at ratios between 2:1 and 3:1 (Al:Si). However, they used much more concentrated solutions of Al and Si (1 M Al chloride and 1 M sodium metasilicate) to prepare the aluminosilicate gel that was subsequently collected and dried for analysis. Whether similar aluminosilicates species are present in the more dilute Al and Si solutions prepared here is not known, but a mixture of aluminosilicate species is certainly possible. Notably, aluminosilicates formed *in situ* in cellular lysosomes of the digestive gland in water snails (*Lymnaea stagnalis*), are of the same size and Al:Si ratio as those formed here. These were proposed to be allophane material and similar in structure to protoimogolite [7]. The lack of phase identification of the ASP material reported here is not too surprising: it is a nanoparticle with only short-to-medium range order. Its small size (~ 4 nm) and given crystalline structure suggest that it may be an early species (e.g. initial formed unit or precursor) for subsequent and distinct aluminosilicate formation as seen by White et al. [7], and Wilson et al., [34]. 

We considered the possibility that ASP identified here by TEM and STEM was formed during sample preparation (i.e. there was an alteration in the original structure during drying of the samples prior to or during TEM/STEM analysis). Certainly, there was some variability in the lattice spacing. However, we don’t believe this to be the case, as findings were comparable between the aqueous methods used here and microscopy, with respect to particle sizes of ASP and HSP. Subtler changes at the level of TEM/STEM may have occurred but in no way change the overall message of this work. Unfortunately, the dilute solutions used here were not suitable for meaningful analysis by NMR or by direct light scattering methods. An alternative approach could be to use the plunge freezing method recently reported by Hondow et al [35], which should allow closer imaging to the hydrated-dispersed state, although even this is indirect. 

Doucet et al. [36] have previously reported on the interaction between monomeric silica (< 2 mM Si) and polyhydroxy Al that form colloidal hydroxyaluminosilicates (HAS). They reported that HAS was not formed in solutions in which Al hydroxide precipitation was not predicted, suggesting that HAS formation is dependent on the prior formation of hydroxy-aluminum species [11,36]. Taken together with the work herein, these studies confirm that for strong interaction to occur between silica and Al(III) at neutral pH, at least one species has to be polymeric: i.e. polymeric silica will bind monomeric Al(III) ions (as shown here) and monomeric silica will bind polyhydroxy Al strongly (as reported by Doucet et al. [36]), and both interactions may result in the formation of secondary aluminosilicates.

The biological relevance of the above findings is not immediately clear, as monomeric silica appears to be *exclusively* absorbed in the mammalian gut [13] and thus polymeric silica would have to be formed *in vivo* to be effective in binding Al(III) ions in the presence of biological ligand: i.e. there needs to be cellular accumulation and polymerization of monomeric silica for it to have Al binding activity *in vivo*. The accumulation and controlled polymerization (bio-mineralization) of monomeric silica, has been described in diatoms and sponges, but not in mammals [37–40]. However, White and colleagues have described, in the freshwater snail *Lymnaea stagnalis*, the intracellular accumulation of Si and its co-localization with Al(III) (as nano-sized HAS) in response to Al-induced behavioral toxicity, as a mechanism for the detoxification of Al(III) [7,8]. It is not clear if similar mechanisms exist in higher life-forms (mammals) to deal with Al(III) toxicity, but the ubiquity and abundance of both elements in the natural environment lead us to suggest that such a mechanism could be widespread. Indeed, the co-localization of Al and Si in lipofuscin granules of the brains of dementia patients and the increased excretion (or reduced re-absorption) of Al(III) with ingestion of high dose of monomeric silica, as outlined in the Introduction, may provide some evidence for this in mammals [41,42]. There may also be environmental niches that allow this kind of chemistry to proceed, creating aluminosilicates from polymeric silica origins [43,44].

## Conclusion

The findings from our studies suggest that the *in vitro* interaction between dilute solutions of high Al-affinity silica polymer (HSP) and Al(III) ions results in the formation of crystalline Al-rich silica nanoparticles (ASP). The estimated Al:Si ratio (2,3) and small size (~ 4 nm) suggest ASP as an early species for subsequent aluminosilicate formation. Moreover, HSP competes very effectively with human apotransferrin for aluminum ions and thus ASP may act as a sink or storage for Al(III) ions in terms of metabolic and certain environmental processes. 

## Supporting Information

Figure S1 **TEM analysis of the high Al-affinity silica polymer.**
(DOC)

## References

[1] Klein C (1993) Reviews in mineralogy: health effects of mineral dust. In: Guthrie GD Jr, Mossman BT Rocks, minerals, and a dusty world. Washington DC: Bookcrafters Inc. p 8.

[2] Parfitt RL (2009) Allophane and imogolite: role in soil biogeochemical processes. Clay Minerals 44: 135-155. doi:10.1180/claymin.2009.044.1.135.

[3] Yang XY, Léonard A, Lemaire A, Tian G, Su BL (2011) Self-formation phenomenon to hierarchically structured porous materials: design, synthesis, formation mechanism and applications. Chem Commun (Camb) 47: 2763-2786. doi:10.1039/c0cc03734f. PubMed: 21246107. 21246107

[4] Brown SH (2010) Zeolites in catalysis: handbook of green chemistry. New Jersey: Wiley & Sons.

[5] Godoi RH, Braga DM, Markarorska Y, Alfoldy B, Carvalho Filho MA et al. (2008) Inhable particulate matter from lime industries: chemical composition and deposition in human respiratory tract. Atmospheric Environ 42: 7027-7033. doi:10.1016/j.atmosenv.2008.07.002.

[6] Lomer MC, Thompson RP, Powell JJ (2002) Fine and ultrafine particles of the diet: influence on the mucosal immune response and association with Crohn’s disease. Proc Nutr Soc 61: 123-130. doi:10.1079/PNS2001134. PubMed: 12002786. 12002786

[7] White KN, Ejim AI, Walton RC, Brown AP, Jugdaohsingh R et al. (2008) Avoidance of aluminum toxicity in freshwater snails involves intracellular silicon−aluminum biointeraction. Environ Sci Technol 42: 189-194. 10.1021/es7028608 18409657

[8] Desouky M, Jugdaohsingh R, McCrohan CR, White KN, Powell JJ (2002) Aluminum-dependent regulation of intracellular silicon in the aquatic invertebrate *Lymnaea* *stagnalis* . Proc Natl Acad Sci U S A 99: 3394-3399. doi:10.1073/pnas.062478699. PubMed: 11891333. 11891333 PMC122534

[9] Flaten TP (2001) Aluminium as a risk factor in Alzheimer’s disease, with emphasis on drinking water. Brain. Res Bull 55: 187-196. doi:10.1016/S0361-9230(01)00459-2. 11470314

[10] Exley C (1999) A molecular mechanism of aluminium-induced Alzheimer’s disease? J Inorg Biochem 76: 133-140. doi:10.1016/S0162-0134(99)00125-7. PubMed: 10612066. 10612066

[11] Exley C (2011) Reflections upon and recent insight into the mechanism of formation of hydroxyaluminosilicates and the therapeutic potential of silicic acid. Coord Chem Rev 256: 82-88.

[12] Swaddle TW (2001) Silicate complexes of aluminium(III) in aqueous systems. Coord Chem Rev 219: 665-686.

[13] Jugdaohsingh R, Reffitt DM, Oldham C, Day JP, Fifield LK et al. (2000) Oligomeric but not monomeric silica prevents aluminum absorption in humans. Am J Clin Nutr 71: 944-949. PubMed: 10731501. 10731501 10.1093/ajcn/71.4.944

[14] Edwardson JA, Moore PB, Ferrier IN, Lilley JS, Newton GWA et al. (1993) Effect of silicon on the gastrointestinal absorption of aluminium. Lancet 342: 211−212. doi:10.1016/0140-6736(93)92301-9. 8100932

[15] Birchall JD, Exley C, Chappell JS, Phillips MJ (1989) Acute toxicity of aluminium to fish eliminated in silicon-rich acid waters. Nature 338: 146-148. doi:10.1038/338146a0.

[16] Bellia P, Birchall JD, Roberts NB (1996) The role of silicic acid in the renal excretion of aluminium. Ann Clin Lab Sci 26: 227-233. PubMed: 8726215. 8726215

[17] Farmer VC, Lumsdon DG (1994) An assessment of complex formation between aluminium and silicic acid in acidic solutions. Geochim Cosmochim Acta 58: 3331-3334. doi:10.1016/0016-7037(94)90088-4.

[18] Anseau MR, Leung JP, Sahai N, Swaddle TW (2005) Interactions of silicate ions with zinc(II) and aluminum(III) in alkaline aqueous solution. Inorg Chem 44: 8023-8032. doi:10.1021/ic050594c. PubMed: 16241152. 16241152

[19] Iler RK (1979) The chemistry of silica: solubility, polymerisation, colloid and surface properties, and biochemistry. New York: John Wiley & Sons.

[20] Taylor PD, Jugdaohsingh R, Powell JJ (1997) Soluble silica with high affinity for aluminum under physiological and natural conditions. J Am Chem Soc 119: 8852-8856. doi:10.1021/ja964476n.

[21] Jugdaohsingh R, Kinrade SD, Powell JJ (2008) Is there a Biochemical Role for Silicon? In: Collery P Maynard I Theophanides T Khassanova L Collery T Metal Ions in Biology and Medicine Vol X. Paris:John Libbey Eurotext pp. 45-55.

[22] Dobbie JW, Smith MJB (1982) Silicate nephrotoxicity in the experimental animal: the missing factor in analgesic nephropathy. Scott Med J 27: 10-16. PubMed: 6278583. 6278583 10.1177/003693308202700104

[23] Vanhaeringen B, Delange F, Vanstokkum I, Srai SKS, Evans RW et al. (1995) Dynamic structure of human serum transferrin from transient electric birefringence experiments. Proteins:_Structure Function and Genetics 23: 233-240. doi:10.1002/prot.340230212. 8592704

[24] Dietzel M (2000) Dissolution of silicates and the stability of polysilicic acid. Geochim Cosmochim Acta 64: 3275-3281. doi:10.1016/S0016-7037(00)00426-9.

[25] Iler RK (1982) Colloidal components in solutions of sodium silicate. In: American Chemical Society, editor. Soluble silicates 194 194 ACS Symp Ser. pp. 95-114.

[26] Hansen PL, Brydson R, McComb DW, Richardson I (1994) EELS fingerprint of Al-coordination in silicates. Microsc Microanal Microstruct 5: 173-182. doi:10.1051/mmm:0199400503017300.

[27] Fatemi SJ, Kadir FH, Moore GR (1991) Aluminium transport in blood serum. Binding of aluminium by human transferrin in the presence of human albumin and citrate. Biochem J 280: 527-532. PubMed: 1747128. 1747128 10.1042/bj2800527 PMC1130580

[28] Trapp GA (1983) Plasma aluminum is bound to transferrin. Life Sci 33: 311-316. doi:10.1016/0024-3205(83)90505-2. PubMed: 6410138. 6410138

[29] Cochran M, Coates J, Neoh S (1984) The competitive equilibrium between aluminium and ferric ions for the binding sites of transferrin. FEBS Lett 176: 129-132. doi:10.1016/0014-5793(84)80926-6. PubMed: 6489514. 6489514

[30] Cochran M, Cochran M, Coates JH, Kurucsev T (1987) Direct spectrophotometric determination of the two site binding of aluminum to transferrin. Life Sci 40: 2337-2341. doi:10.1016/0024-3205(87)90507-8. PubMed: 3586862. 3586862

[31] Jugdaohsingh R (1999) Soluble silica and aluminium bioavailability. PhD thesis University of London.

[32] Hajimohammadi A, Provis JL, van Deventer JS (2008) One-Part Geopolymer Mixes from Geothermal Silica and Sodium Aluminate. Ind Eng Chem Res 47: 9396-9405. doi:10.1021/ie8006825.

[33] Dietzel M, Usdowski E, Hoefs J (1992) Chemical and 13 C/^12^C- and ^18^O/^16^O-isotope evolution of alkaline drainage waters and the precipitation of calcite. Appl Geochem 7: 177-184

[34] Wilson MA, McCarthy SA, Fredericks PM (1986) Structure of poorly-ordered aluminosilicates. Clay Minerals 21: 879-897. doi:10.1180/claymin.1986.021.5.03.

[35] Hondow N, Brydson R, Wang P, Holton MD, Brown MR et al. (2012) Quantitative Characterization of Nanoparticle Agglomeration within Biological Media. J Nanopart Res 14: 977. doi:10.1007/s11051-012-0977-3.

[36] Doucet FJ, Schneider C, Bones SJ, Kretchmer A, Moss I et al. (2001) The formation of hydroxyaluminosilicates of geochemical and biological significance. Geochim Cosmochim Acta 65: 2461-2467. doi:10.1016/S0016-7037(01)00571-3.

[37] Cha JN, Shimizu K, Zhou Y, Christiansen SC, Chmelka BF et al. (1999) Silicatein filaments and subunits from a marine sponge direct the polymerization of silica and silicones *in* *vitro* . Proc Natl Acad Sci U S A 96: 361-365. doi:10.1073/pnas.96.2.361. PubMed: 9892638. 9892638 PMC15141

[38] Shimizu K, Cha J, Stucky GD, Morse DE (1998) Silicatein α: Cathepsin L-like protein in sponge biosilica. Proc Natl Acad Sci U S A 95: 6234–6238. doi:10.1073/pnas.95.11.6234. PubMed: 9600948. 9600948 PMC27641

[39] Kröger N, Deutzmann R, Bergsdorf C, Sumper M (2000) Species-specific polyamines from diatoms control silica morphology. Proc Natl Acad Sci U S A 97: 14133-14138. doi:10.1073/pnas.260496497. PubMed: 11106386. 11106386 PMC18883

[40] Kröger N, Deutzmann R, Sumper M (1999) Polycationic peptides from diatom biosilica that direct silica nanosphere formation. Science 286: 1129-1132. doi:10.1126/science.286.5442.1129. PubMed: 10550045. 10550045

[41] Tokutake S (1997) Accumulation of aluminum and silicon in lipofuscin granules suggests retardation of blood–brain barrier function by aging. Ann N Y Acad Sci 826: 510-512. doi:10.1111/j.1749-6632.1997.tb48515.x. PubMed: 9329735. 9329735

[42] Tokutake S, Nagase H, Morisaki S, Oyanagi S (1995) Aluminium detected in senile plaques and neurofibrillary tangles is contained in lipofuscin granules with silicon, probably as aluminosilicate. Neurosci Lett 185: 99-102. doi:10.1016/0304-3940(94)11234-A. PubMed: 7746513. 7746513

[43] Teien HC, Kroglund F, Atland A, Rosseland BO, Salbu B (2006) Sodium silicate as alternative to liming-reduced aluminium toxicity for Atlantic salmon (*Salmo* *salar* L.) in unstable mixing zones. Sci Total Environ 358: 151-163. doi:10.1016/j.scitotenv.2005.09.052. PubMed: 16225906. 16225906

[44] Wonisch H, Gerard F, Dietzel M, Jaffrain J, Nestroy O et al. (2008) Occurrence of polymerized silicic acid and aluminum species in two forest soil solutions with different acidity.Geoderma 144: 435-445. doi:10.1016/j.geoderma.2007.11.022.

