# Supplementary material for: High-Aluminum-Affinity Silica Is a Nanoparticle That Seeds Secondary Aluminosilicate Formation
Source: PLoS One. 2013 Dec 13;8(12):e84397. doi: 10.1371/journal.pone.0084397 (PMC3862809; doi:10.1371/journal.pone.0084397)
Supplement: Figure S1 — TEM analysis of the high Al-affinity silica polymer. (DOC) [file pone.0084397.s001.doc]

**FIGURE S1.**


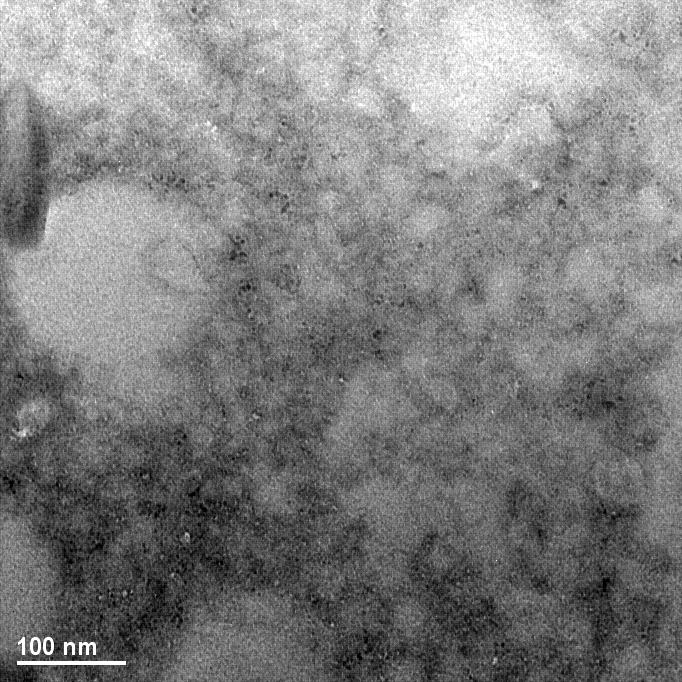

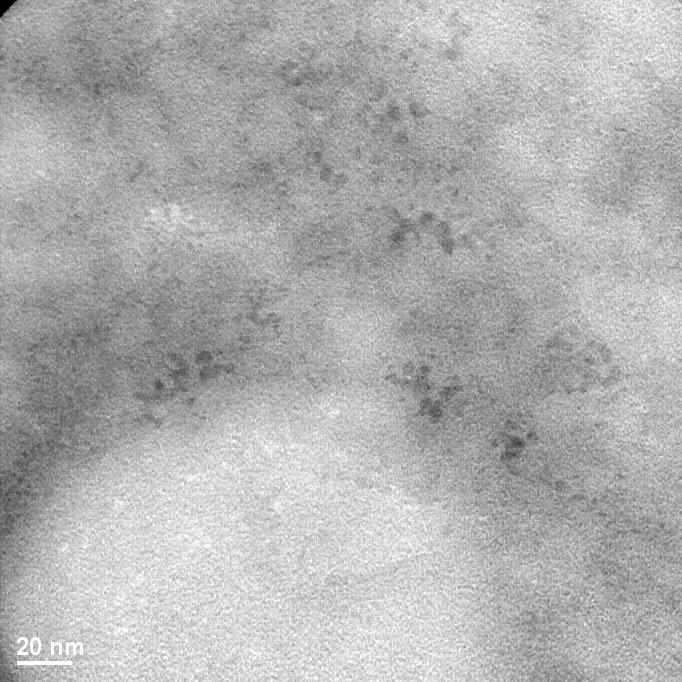


**A**

**B**


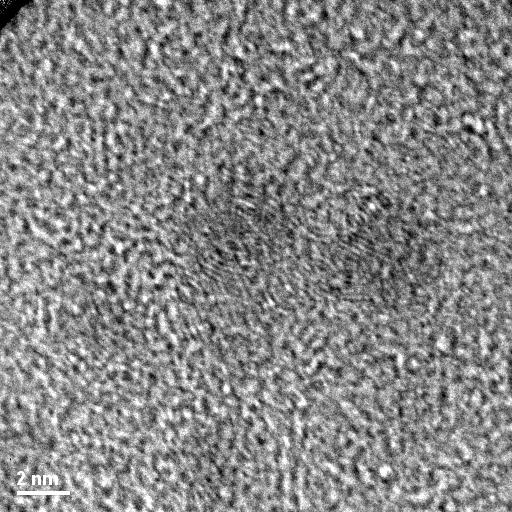


**C**

**Figure S1. TEM analysis of the high Al-affinity silica polymer.** Transmission electron microscopy analysis of the air-dried high-aluminum-affinity silica polymer (HSP)-containing solution (320 µM Si, pH 7.2) in the absence of added Al(III). An amorphous silica gel with mottled appearance and dark particulate matter a few nm in diameter (A & B) was observed under low magnification in bright field TEM. High magnification TEM (C) showed these particulates to be < 5nm in size with some cystallinity. EDX analysis showed these to be rich in Si and O (data not shown).
